# Present Scenario of Circular RNAs (circRNAs) in Plants

**DOI:** 10.3389/fpls.2019.00379

**Published:** 2019-04-02

**Authors:** Wei Zhao, Shanshan Chu, Yongqing Jiao

**Affiliations:** ^1^Collaborative Innovation Center of Henan Grain Crops, College of Agronomy, Henan Agricultural University, Zhengzhou, China; ^2^Key Laboratory of Biology and Genetic Improvement of Oil Crops, Ministry of Agriculture, Oil Crops Research Institute, Chinese Academy of Agricultural Sciences, Wuhan, China

**Keywords:** circular RNA, circRNA, non-coding RNA, bioinformatics, review

## Abstract

Circular RNAs (circRNAs) are new endogenous non-coding RNA family members that arise during pre-mRNA splicing in a reversed order in which the 3′ and 5′ ends are covalently closed. Compared to the comprehensive investigation of circRNAs in animals, circRNA research in plants is still in its infancy. Genome-wide identification and characterization of circRNAs have recently been performed in several plant species. CircRNAs are ubiquitously expressed and abundant in plants. The expression of circRNAs is often dependent on cell-type, tissue, and developmental stage, and it is particularly stress-inducible in plants. CircRNAs might play important roles in various biological processes in plants, including development and the response to biotic and abiotic stresses. Here, we review the current literature and provide a brief overview of circRNAs and their research status in plants, as well as the bioinformatic tools and database resources for circRNA analysis.

## Introduction

**Figure 1:**
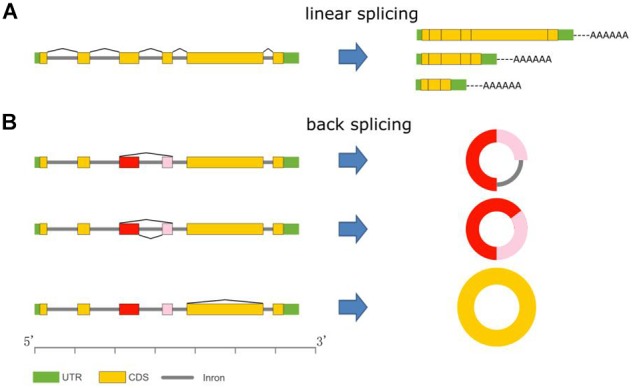
CircRNA biogenesis. **(A)** mRNA variants via alternative linear splicing. All mRNAs have polyA tails. **(B)** CircRNA variants formation via backsplicing.

Unlike the better-known linear mRNAs formed by linear splicing, circular RNAs (circRNAs) are novel members of the non-coding RNA family and are generated during post-transcriptional processes via backsplicing of precursor messenger RNAs (pre-mRNAs) (Figure 1A,B) (Jeck et al., 2013). CircRNAs could be derived from any genomic location, such as exonic, intronic, and intergenic regions (Chen, 2016). In animals, circRNAs are widespread and are the predominant isoform of exons originating from protein-coding genes spliced in a non-canonical order (Salzman et al., 2012; Westholm et al., 2014; Rybak-Wolf et al., 2015). Unlike normal linear RNAs, circRNAs form covalently closed loop structures with neither 5′–3′ polarities nor polyadenylated tails. Thus, circRNAs are resistant to RNase R, which is a strong 3′–5′ exoribonuclease that is able to efficiently degrade linear RNAs. Therefore, circRNAs can be segregated and enriched from eukaryotic total RNAs by RNase R digestion (Suzuki and Tsukahara, 2014; Chen and Yang, 2015).

Although the existence of circular transcripts has been observed for decades, such as the hepatitis δ virus and some plant viroids, circRNAs were typically regarded as rare byproducts of errant splicing during mRNA processing in eukaryotic cells (Sanger et al., 1976; Kos et al., 1986; Memczak et al., 2013). Due to the recent developments in high-throughput deep sequencing technology, exonuclease-based enrichment strategies, and novel bioinformatic tools, numerous circRNAs have been discovered and identified in many organisms, such as humans (Jeck et al., 2013), mouse (Fan et al., 2015), zebrafish (Shen et al., 2017), archaea (Danan et al., 2012), rice (Lu et al., 2015), and *Arabidopsis* (Ye et al., 2015), leading to the consensus that circRNAs are ubiquitous and abundant in eukaryotes.

Plant circRNAs possess features that differ from animal circRNAs. For example, reverse complementary elements, which are important for circularization, are enriched in the flanking introns of circRNAs in animals (Jeck and Sharpless, 2014). In contrast, in plants, most of the identified circRNAs contain comparatively fewer repetitive and reverse complementary sequences in the flanking introns that bracket the circRNAs (Lu et al., 2015; Ye et al., 2015). Additionally, in animals, certain circRNAs have been reported to act as miRNA sponges to regulate the expression of target genes. However, studies of circRNAs in plants have not implied the potential suitability of circRNAs as miRNA sponges (Hansen et al., 2013; Memczak et al., 2013; Westholm et al., 2014). Thus, plant circRNAs might possess different mechanisms of biogenesis and have different functional roles from animal circRNAs.

In this review, we present a concise and up-to-date overview of circRNAs in plants. Particularly, we focus on the abundance and expression patterns of circRNAs in various plant species and then discuss the available bioinformatic resources that can be used to characterize circRNAs based on high-throughput sequencing data. Finally, the potential functionality of circRNAs in plants is explored.

## CircRNA Abundance in Plants

It is challenging to separate circRNAs from other RNAs, such as miRNA and mRNA, based on size or electrophoretic mobility. Due to the lack of a free polyadenylated tail, circRNAs have evaded recognition by poly (A) enrichment approaches. Thus, although circRNAs have been observed in eukaryotic cells for decades, it has not been possible to comprehensively evaluate them. Recent developments in high-throughput deep sequencing coupled with exonuclease-based enrichment strategies and computational approaches have resulted in the identification of thousands of circRNAs in animals, including in *Drosophila* (Westholm et al., 2014), humans (Salzman et al., 2012), mouse (Fan et al., 2015), and zebrafish (Shen et al., 2017).

Similarly, limited studies on higher plants have revealed that circRNAs are also widespread and abundant in plant species (Table 1). The genome-wide identification of plant circRNAs was first performed in *Oryza sativa* and *Arabidopsis thaliana*. Ye et al. (2015) identified 12,037 circRNAs from the root and shoot tissues of *O. sativa* and 6,012 circRNAs from the leaves of *A. thaliana*. Furthermore, Ye et al. (2017) also identified 2,806 circRNAs in the root tissues of rice seedlings. Lu et al. (2015) reported 2,354 circRNAs in the panicles and mature leaves of rice at different flowering time stages. In addition to the two well-known model plants, circRNAs have also been identified from the other monocotyledon and dicotyledon species, such as barley, tomato, wheat, soybean, and kiwifruit (Table 1). Darbani et al. (2016) reported 62 circRNAs in the leaves and immature seeds of barley, while Zuo et al. (2016) identified 854 circRNAs from mesocarp samples at the mature green stage of tomato. Additionally, Tan et al. (2017) also identified 796 circRNAs from the fruits of tomato. Using an RNase R-treated enrichment approach, Wang et al. (2017) detected 88 circRNAs in the leaves of wheat seedlings. Zhao et al. (2017a) identified 5,372 circRNAs in the leaves, stems, and roots of soybean seedlings, and another study also identified 5,367 circRNAs associated with resistance to defoliating insects in soybean leaves (Zhao et al., 2017b). Wang et al. (2017) identified 3,582 circRNAs from the leaf, root, and stem tissues of four kiwifruit materials originating from three species belonging to the genus *Actinidia*. Chen et al. (2018) detected 2,804 circRNAs in the seedling leaves of maize. In summary, an abundance (95,143) of circRNAs has been identified from various plant species (Chu et al., 2018); however, regrettably, only a few circRNAs have been verified by experimental approaches. With the development of high-throughput technology and new bioinformatic tools, we anticipate that more circRNAs will be identified in plants in the future.

**Table 1:** Studies showing the genome-wide identification of circRNAs in plants.

No	Year	Publication Details and Reference No.	Plant species	Number of circRNAs	Tissues/Developmental Stages	Approach	Stimuli/Biological Process	
							Abiotic Stress	Biotic Stress
1	2015	Ye et al., 2015	Oryza sativa	12,037	Roots and shoots the following time	rRNA-depleted RNA-Seq	Pi-starvation	
		New Phytol.	(Rice)					
2	2015	Lu et al., 2015	Oryza sativa	2,354	Panicles and mature leaves at the flowering time	rRNA-depleted/RNase R-treated RNA-Seq		
		RNA	(Rice)					
3	2017	Ye et al., 2017	Oryza sativa	2,806	Roots of seedlings	rRNA-depleted/RNase R-treated RNA-Seq		
		RNA Biol.	(Rice)					
4	2015	Ye et al., 2015	Arabidopsis thaliana	6,012	Leaves of 3–4 weeks old plants	rRNA-depleted RNA-Seq	Light	
		New Phytol.						
5	2016	Sun et al., 2016	Arabidopsis thaliana	970	NA	rRNA-depleted RNA-Seq		
		FEBS						
6	2016	Darbani et al., 2016	Hordeumvulgare	47	Leaves and immature seeds at the growth stage 18 ± 2 days after anthesis	rRNA-depleted RNA-Seq		
		Frontiers in Plant Science	(Barley)					
7	2016	Zuo et al., 2016	Solanumlycopersicum	854	Mesocarp samples at mature green stage	rRNA-depleted RNA-Seq	Chilling	
		BBRC	(tomato)					
8	2017	Wang et al., 2017	Triticum aestivum	88	Leaves of seedlings	rRNA-depleted/RNase R-treated RNA-Seq	Dehydration	
		Frontiers in Plant Science	(Wheat)					
9	2017	Zhao et al., 2017b	Soybean	5,367	Leaves at mature green stage	rRNA-depleted RNA-Seq		Cotton bollworm
		OCS						
10	2017	Wang et al., 2017	Kiwifruit	3,582	Leaf, root, and stem tissues of seedlings	rRNA-depleted RNA-Seq		*Pseudomonas syringae* pv. *actinidiae*
		Frontiers in Plant Science						
11	2017	Zhao et al., 2017a	Soybean	5,372	Leaf, root, and stem tissues of seedlings	rRNA-depleted RNA-Seq		
		Sci. Rep.						
12	2017	Tan et al., 2017	Solanumlycopersicum	796	Fruits	rRNA-depleted RNA-Seq		
		Sci. Rep.	(tomato)					
13	2018	Chen et al., 2018	Maize	2804	B73 seedling leaves	rRNA-depleted RNA-Seq		
		New Phytol.						

## Features of Plant circRNAs

The size of circRNAs ranges from smaller than 100 nt to larger than 4 kb. For example, the majority of human circRNAs are a few hundred nucleotides in length, while soybean circRNAs are mainly between 150 and 600 bp, and only a few are greater than 2 kb (Jeck et al., 2013; Zhang et al., 2014; Zhao et al., 2017a).

A conserved feature in animals and plants is that the genesis of circRNAs depends on RNA polymerase II-mediated transcription and backsplicing reactions of pre-mRNAs (Sun et al., 2016). Previous studies have demonstrated that the reverse complementary sequences were significantly enriched in the flanking introns bracketing circRNAs and that these short intronic repeat sequences could facilitate circRNA production in animals (Jeck et al., 2013; Chen and Yang, 2015; Chen, 2016). For example, in humans, circularized exons are typically bracketed by long introns, which contain abundant *Arthrobacter luteu* (Alu) elements (Jeck et al., 2013). However, there are comparatively fewer of these repetitive elements in plant circRNAs. For example, the proportion of reverse complementary sequences was only 6.2, 2.7, and 0.3% in the intronic sequences flanking exonic circRNAs in rice, soybean, and *A. thaliana*, respectively (Lu et al., 2015; Ye et al., 2015; Zhao et al., 2017a). In maize, sequences related to LINE1-like elements (LLEs) and their Reverse Complementary Pairs (LLERCPs) are significantly enriched in the flanking regions of circRNAs, which indicates that transposons may be involved in the formation of circRNAs in plants (Wang et al., 2017).

CircRNAs are generated when the pre-mRNA splicing machinery backsplices to join a down-stream splice donor to an upstream splice acceptor. The 3′ and 5′ ends normally present in a linear mRNA molecule have been joined together covalently in circRNAs. The U2-dependent spliceosome is responsible for the splicing of the vast majority of introns in both plants and animals, with GT and AG terminal dinucleotides at their 5′ and 3′ termini, respectively (Szczesniak et al., 2013). However, the mechanisms of selection for certain sequences to circularize by spliceosomes are poorly characterized. The analysis of splice signals of circRNAs in humans has revealed that most of the exonic circRNAs contain canonical GT/AG splicing signals, while some also harbor non-GT/AG splicing signals (Ye et al., 2017). In plants, the splice signals of circRNAs differ between monocot and dicot species. In rice, only a small portion (7.3%) of circRNAs possess canonical GT/AG (CT/AC) splicing signals, and a large number of circRNAs share diverse non-GT/AG splicing signals, such as GC/GG, CA/GC, GG/AG, GC/CG, and CT/CC (Ye et al., 2017). In *A. thaliana*, among the 803 identified circRNAs with the GT-AG signal, the majority showed the canonical splicing signal GT/AG, while only 9 circRNAs were generated from non-canonical splicing signals (Sun et al., 2016). However, these diverse splice signal patterns should be further verified in more plant species.

CircRNAs are conserved and have various isoforms that are generated by alternative circularization in plants (Figure 1B). In *O. sativa* and *A. thaliana*, 6,074 and 5,152 circRNA-host genes could generate exonic circRNAs, of which 12.2 and 14.5% constitute orthologous genes. Furthermore, over 300 orthologous circRNA-host genes could generate circRNAs from a similar position (Ye et al., 2015).

Alternative circularization constitutes another circRNA formation mechanism that can generate a variety of additional circRNAs from one gene (Zhang et al., 2014). A previous study demonstrated that over 50% of gene loci produced circRNAs through alternative circularization in human and mouse cells (Zhang et al., 2014). Similarly, in rice, a large number of circRNA isoforms are produced by the same locus, and over half of the circRNAs in rice were generated by alternative circularization (Ye et al., 2015). For example, the gene *LOC_Os11g02080* was predicted to generate 41 isoforms, while the gene *LOC_Os12g02040* was predicted to generate 38 isoforms, which were further validated by the successful sequencing of reverse transcription (RT)-PCR products (Ye et al., 2015).

## Bioinformatic Resources for Plant circRNAs

The advances in high-throughput deep sequencing technology have enabled scientists to generate millions of sequencing reads in a short time period. In response to the mass generation of RNA sequencing (RNA-Seq) data, new computational algorithms for the precise and efficient identification of circRNAs have been developed (Szabo et al., 2015). Several different bioinformatic tools, such as circRNA finder (Westholm et al., 2014), CIRCexplorer (Zhang et al., 2014), CIRI (Gao et al., 2015), find circ (Memczak et al., 2013), Mapsplice (Wang et al., 2010), PcircRNA_finder (Chen L. et al., 2016), and circseq-cup (Ye et al., 2017), have been developed specifically for this purpose (Table 2). However, these bioinformatic tools perform differently in terms of precision and sensitivity when identifying circRNAs from RNA-Seq data and also differ in computational costs. PcircRNA_finder was specially developed for plant circRNA identification and provided a more comprehensive, sensitive, and precise prediction method for plant circRNAs, while the other algorithms were established for animals (Chen L. et al., 2016). Hansen et al. (2016) comparatively analyzed circRNA prediction tools. They found that different tools could produce highly divergent results with high false positive ratios; however, combining the output of the different tools could reduce the false positive fraction significantly (Hansen et al., 2016). Zeng et al. (2017) also comprehensively evaluated different circRNA detection tools using four different datasets, including a positive dataset, background dataset, mixed dataset, and real datasets. Generally, CIRI, CIRCexplorer, and KNIFE perform better in terms of achieving a balance between precision and sensitivity. These comparison results proved valuable for improving algorithms and have provided useful guidelines for current algorithms used for data interpretation by researchers.

**Table 2:** An overview of the bioinformatics tools available for the prediction of circRNAs.

Tools	Latest version	Mapper	Websites	Reference
circRNA finder	N/A	STAR	https://github.com/orzechoj/circRNA_finder	Westholm et al., 2014
CIRCexplorer	1.1.10	Bowtie1 and 2	https://github.com/YangLab/CIRCexplorer	Zhang et al., 2014
CIRI	1.2	Bwa	https://sourceforge.net/projects/ciri/files/	Gao et al., 2015
find circ	v2	Bowtie2	https://github.com/marvin-jens/find_circ	Memczak et al., 2013
Mapsplice	2.2.1	Bowtie1	http://www.netlab.uky.edu/p/bioinfo/MapSplice2	Wang et al., 2010
circseq-cup	1.0	STAR	http://ibi.zju.edu.cn/bioinplant/tools/circseq-cup.htm	Ye et al., 2017
KNIFE	1.4	Bowtie, Bowtie2	https://github.com/lindaszabo/KNIFE	Szabo et al., 2015
Segemehl	0.2.0	Segemehl	http://www.bioinf.uni-leipzig.de/Software/segemehl/	Hoffmann et al., 2014
UROBORUS	0.0.2	Bowtie, Bowtie2, tophat2	http://uroborus.openbioinformatics.org/en/latest/	Song et al., 2016

Increasing numbers of circRNA datasets have been produced, which has urgently necessitated the efficient organization and management of these datasets. Currently, several animal circRNA databases have been established, such as circ2Traits (Ghosal et al., 2013), nc2Cancer (Chen et al., 2015), circBase (Glažar et al., 2014), starBase v2.0 (Li et al., 2014), CircNet (Liu Y.C. et al., 2016), deepBase v2.0 (Zheng et al., 2016), CircInteractome (Dudekula et al., 2016), and circRNADb (Chen X. et al., 2016) (Table 3). Comparatively, only one plant-specific circRNA database, PlantcircBase^1^, has been created recently (Chu et al., 2017). PlantcircBase records published and unpublished circRNAs with universal identifiers from different plants species, including *O. sativa*, *A. thaliana*, *Zea mays* (*Z. mays*), *Solanum lycopersicum* (*S. lycopersicum*), *Glycine max* (*G. max*), *Camellia sinensis* (*C. sinensis*), *Gossypium arboreum* (*G. arboreum*), *Gossypium hirsutum* (*G. hirsutum*), *Gossypium raimondii* (*G. raimondii*), *Ptelea trifoliata* (*P. trifoliata*), *Symphytum tuberosum* (*S. tuberosum*), *Triticum aestivum* (*T. aestivum*), and *Hordeum vulgare* (*H. vulgare*). PlantcircBase also provides a visualization of specific circRNA structures, potential interaction networks involving circRNA-miRNA-mRNA in the corresponding species, and validation information by Sanger sequencing. However, more significant information on functional annotation, tissue expression, interaction with other molecules, and phylogenetic conservation are still needed, which would make PlantcircBase a more comprehensive resource for research into plant circRNAs. The development of high-throughput sequencing technologies and the growing availability of various bioinformatic resources will greatly promote circRNA research.

**Table 3:** Public circRNA databases.

Name	Species	Description/Main Features	Website	Reference
circ2Traits	*H. sapiens*	A comprehensive database for circRNA potentially associated with disease and traits in humans.	http://gyanxet-beta.com/circdb/	Ghosal et al., 2013
		Not plant-specific.		
nc2Cancer	*H. sapiens*	A comprehensive association between ncRNAs and cancer in humans.	http://www.bioinfo.tsinghua.edu.cn/nc2Cancer	Chen et al., 2015
		Not plant-specific.		
circBase	*H. sapiens*,	A comprehensive database of animal circRNAs.	http://www.circbase.org/	Glažar et al., 2014
	M. musculus,	Not plant-specific.		
	D. melanogaster,			
	C. elegans,			
	L. chalumnae,			
	L. menadoensis			
starBase v2.0	*H. sapiens*,	A comprehensive database of CLIP-Seq experimentally supported miRNA-ceRNA, miRNA-ncRNA and protein-RNA interaction networks.	http://starbase.sysu.edu.cn	Li et al., 2014
	M. musculus,	Not plant-specific.		
	C. elegans			
CircNet	*H. sapiens*	A database of tissue-specific circRNA expression profiles and circRNA-miRNA-gene regulatory networks in humans.	http://circnet.mbc.nctu.edu.tw/	Liu Y.C. et al., 2016
		Not plant-specific.		
deepBase v2.0	*H. sapiens*,	An integrated knowledge database with comprehensive collection and annotation of non-coding RNAs including small RNAs, LncRNAs, and circRNAs.	http://biocenter.sysu.edu.cn/deepBase/	Zheng et al., 2016
	M. musculus,	Not plant-specific.		
	C. elegans			
CircInteractome	*H. sapiens*	Interaction of circRNAs and proteins and microRNAs in humans.	http://circinteractome.nia.nih.gov	Dudekula et al., 2016
		Not plant-specific.		
circRNADb	*H. sapiens*	Human circular RNAs with protein-coding annotations.	http://reprod.njmu.edu.cn/circrnadb/circRNADb.php	Chen X. et al., 2016
		Not plant-specific.		
PlantcircBase 3.0	*O. sativa*	A comprehensive database of plant circRNAs.	http://ibi.zju.edu.cn/plantcircbase/index.php	Chu et al., 2017, 2018
	A. thaliana	Plant-specific.		
	Z. mays			
	S. lycopersicum			
	G. max			
	C. sinensis			
	G. arboreum			
	G. hirsutum			
	G. raimondii			
	P. trifoliata			
	S. tuberosum			
	T. aestivum			
	H. vulgare			

## Expression Patterns of Plant circRNAs

CircRNAs usually exhibit specific cell-type, tissue, and developmental stage expression patterns in animals (Westholm et al., 2014; Fan et al., 2015). For example, in *Drosophila*, circRNAs originate from neural genes and exhibit enhanced accumulation in neural tissues (Westholm et al., 2014). Using single-cell universal poly(A)-independent RNA sequencing technology, Fan et al. (2015) discovered 2,891 circRNAs in mouse preimplantation embryos, of which a large proportion showed developmental-specific expression patterns.

In plants, circRNAs also exhibit specific expression patterns, as observed in animals (Lu et al., 2015; Darbani et al., 2016). Darbani et al. (2016) treated field-grown barley plants with a foliar application of iron or zinc solution and collected the seed transfer cells using laser capture micro-dissection for RNA-Seq analysis. They ultimately identified 62 transfer cell-specific circRNAs and demonstrated that these circRNAs could respond to the foliar application of micronutrients in barley (Darbani et al., 2016). Lu et al. (2015) validated 30 rice circRNAs experimentally, of which three and four circRNAs showed panicle-specific and leaf-specific expression, respectively.

Plant circRNAs also exhibit stress-inducible expression patterns. Ye et al. (2015) found that 27 circRNAs were differentially expressed under phosphate-sufficient -sufficient and -starvation conditions in rice. Additionally, by comparing the expressions of the circRNAs between low- and high-light stress conditions, they found that many circRNAs were specifically expressed following high-light treatment in *Arabidopsis* (Ye et al., 2015). In tomato, approximately 19% (163/854) of circRNAs exhibited chilling responsive expression patterns (Zuo et al., 2016). In wheat, 62 circRNAs were differentially expressed in dehydration-stressed seedling leaves (Wang Y. et al., 2016).

In addition to abiotic stress, circRNAs were also reported to be responsive to biotic stresses. For example, in soybean, 199 circRNAs were found to be differentially expressed between resistant and susceptible samples under defoliation damage by cotton bollworm feeding (Zhao et al., 2017b). Another study in kiwifruit revealed that 584 circRNAs were differentially expressed during *Pseudomonas syringae* pv. *actinidiae* (Psa) infection (Wang et al., 2017). These results suggest that circRNAs might play important and diverse functional roles in response to biotic and abiotic stresses in plants.

## Putative Functions of Plant circRNAs

Although the abundance of circRNAs has been recognized, their functions have remained largely unclear. However, accumulating evidence suggests that circRNAs could play important functional roles in various biological processes, such as miRNA binding, protein binding, and transcriptional regulation (Hansen et al., 2013; Memczak et al., 2013; Chen, 2016).

Previous studies in mammals demonstrated that circRNAs can function as miRNA sponges or potent ceRNA molecules to bind specific miRNAs to prohibit them from regulating their target genes (Memczak et al., 2013). For example, in mouse, the sex-determining region Y (Sry) is a highly expressed, testis-specific circRNA that harbors 16 putative binding sites for miR-138 and serves as a miR-138 sponge (Rybak-Wolf et al., 2015). In humans, ciRS-7 (also termed CDR1as), as a circular miR-7 inhibitor, functions as an efficient microRNA sponge. It harbors more than 70 conventional miR-7 binding sites and strongly suppresses miR-7 activity, resulting in increased levels of miR-7 targets (Memczak et al., 2013). However, there is no further evidence to support that miRNA sponge function has been ascribed to the majority of circRNAs in animals. Nevertheless, only 6.6 and 5.0% of circRNAs were predicted to potentially target mimics of miRNAs in rice and *Arabidopsis*, respectively (Ye et al., 2015). Recently, 24 tomato circRNAs and 6 wheat circRNAs were thought to act as miRNA sponges, which requires further validation (Zuo et al., 2016; Wang et al., 2017). Os08circ16564, a rice circRNA, was predicted to harbor the target sites of miR172 and miR810 (Lu et al., 2015). Transgenic analysis for the overexpression of Os08circ16564 showed that the expression level of miR170 did not differ between Os08circ16564-transgenic rice and the control (Lu et al., 2015). It is thus unclear if miRNA inhibition is a general functional aspect of circRNAs in plants and animals—a topic that requires further experimental investigation.

It was previously thought that circRNAs could not produce a natural protein since most of them were not associated with polysomes (Guo et al., 2014). However, some engineered circRNAs with an internal ribosome entry site (IRES) could be translated *in vivo* (Wang and Wang, 2015). Recent research findings in mammals and flies have revealed that endogenous circRNAs containing IRES sequences and ATG could produce proteins. For example, circMbl, a circRNA that exists in the heads of flies, could generate a 37.04 kDa protein (Pamudurti et al., 2017). The Circ-ZNF609 circRNA in humans contains an intact open reading frame (ORF) with start and stop codons that could be translated into a protein in a splicing-dependent and cap-independent manner (Legnini et al., 2017). Moreover, the circ-ZNF609-derived protein plays important functional roles in myogenesis (Legnini et al., 2017). To date, no plant circRNAs have been reported to generate proteins. As research progresses, we expect that the protein coding circRNAs and their potential functions will be revealed in plants.

Previous studies revealed that circRNAs are associated with various physiological and pathophysiological processes, such as insulin biosynthesis and secretion (Xu et al., 2015), neurological diseases (Satoh and Yamamura, 2004; Junn et al., 2009; Lukiw, 2013), degenerative diseases (Ashwal-Fluss et al., 2014; Liu Q. et al., 2016), cardiovascular diseases (Burd et al., 2010; Wang K. et al., 2016; Du et al., 2017), and cancers (Qu et al., 2015; Sand et al., 2016; Shang et al., 2016; Xuan et al., 2016), which indicates that circRNAs might be novel biomarkers for disease diagnosis and therapy in humans. CircRNAs were also reported to serve as a class of aging biomarkers in the central nervous system (CNS) (Westholm et al., 2014). In plants, biomarkers have been widely used for both molecular fundamental research and applied practices in crop breeding (Yang et al., 2011). CircRNAs could also be potential biomarkers in plants due to their unique characteristics, including resistance to degradation, long half-lives, and the ease and specificity of detection (Lai et al., 2018). A recent study revealed that circRNAs can function as bona fide biomarkers of functional exon-skipped AS variants, including in the homeotic MADS-box transcription factor family in *Arabidopsis* (Conn et al., 2017). CircRNAs usually exhibit specific cell-type, tissue, and developmental stage expression patterns, and furthermore, the expression of circRNAs and circRNA isoforms is often induced under diverse environmental stresses, such as low- and high-light stresses, Pi-starvation conditions, low temperature stress, dehydration stress, and chewing injury stress by insects, which suggests that circRNAs might play important roles in plant development or in the response to biotic and abiotic stresses. One subclass of circRNAs in human cells, EIciRNAs (exon–intron circRNAs), was shown to enhance the transcription of the gene from which they were derived through interaction with U1 snRNP and RNA Polymerase II in the promoter region of the circRNA-host gene (Li Z. et al., 2015). Thus, EIciRNAs have the potential to act as transcriptional regulators to induce the expression of circRNA-host genes. In plants, Zhao et al. (2017b) discovered 293 EIcircRNAs, including 183 and 175 in resistant and susceptible samples, under defoliation damage stress by cotton bollworm feeding in soybean, which indicated that EIcircRNAs might participate in the response to chewing injury resistance processes in plants. In addition, some barley circRNAs that are highly expressed in the mitochondria might be involved in micronutrient homeostasis. The overexpression of PSY1-circ1, a circRNA derived from *Phytoene Synthase 1* (*PSY1*) in tomato, resulted in a significant decrease in lycopene and β-carotene accumulation in transgenic tomato fruits, which suggests the involvement of circRNAs in plant development (Tan et al., 2017). However, the detailed mechanisms underlying their regulatory roles in response to biotic and abiotic stresses are still poorly understood.

## Future Perspectives

Interest in the identification and characterization of plant circRNAs is growing. With abundance of circRNAs being identified and new insights into circRNAs generated rapidly, the biogenesis and functionality of circRNAs has become a pertinent research topic. The expression of circRNAs is usually specific to cell-type, tissue, and developmental stage, and is also stress-inducible in both animals and plants, which indicates that circRNAs may represent a new layer of post-transcriptional gene regulation. Evidence in animals indicates that circRNAs are potentially important regulators in various biological processes and are associated with human disorders, including cancers (Li Y. et al., 2015).

CircRNAs potentially represent another level of post-transcriptional gene regulators. Although circRNAs were previously regarded as a novel class of non-coding RNAs, several studies have provided initial evidence for the coding of proteins in animals by certain endogenous circRNAs (Legnini et al., 2017; Pamudurti et al., 2017; Yang et al., 2017). However, the coding potential of plant circRNAs has not been investigated thus far. In the future, elucidating and understanding the functional roles of circRNAs, such as in plant development, the response to biotic and abiotic stresses, and in translation, might constitute the primary research topic in plant circRNAs. Additionally, the differences in circRNA biogenesis between plants and animals would also be an interesting research avenue.

## Data Availability

No datasets were generated or analyzed for this study.

## Author Contributions

WZ and YJ wrote and revised the manuscript. SC helped to prepare the materials and revise the manuscript. All authors read and approved the final manuscript.

## Conflict of Interest Statement

The authors declare that the research was conducted in the absence of any commercial or financial relationships that could be construed as a potential conflict of interest.

## Footnotes

1 http://ibi.zju.edu.cn/plantcircbase/
